# Customer Mistreatment and Venting to Conversational AI: Emotional Exhaustion as Mediator and Trust in Conversational AI as Moderator

**DOI:** 10.3390/bs16040520

**Published:** 2026-03-31

**Authors:** Jialin Cheng, Jingxuan Jiang

**Affiliations:** 1 School of Economics and Management, Zhejiang Sci-Tech University, Hangzhou 310018, China; chengjl050520@zstu.edu.cn; 2 College of Business, Shanghai University of Finance and Economics, Shanghai 200433, China

**Keywords:** customer mistreatment, emotional exhaustion, venting to conversational artificial intelligence (CAI), trust in CAI

## Abstract

Artificial intelligence (AI) technologies, such as service robots, substantially influence frontline employees in the hospitality sector. This study highlights that conversational AI (CAI) may function as a viable outlet for hospitality workers to vent negative work-related issues. This function is particularly relevant because employees in this industry frequently experience customer mistreatment. Grounded in conservation of resources theory, we conceptualize venting to CAI as a resource-replenishing coping strategy triggered by customer mistreatment. Further, we theorize that this relationship is mediated by emotional exhaustion and moderated by trust in CAI, thereby strengthening the indirect effect. We collected and analyzed two-wave data from 394 frontline employees with CAI experience in the hospitality industry. The results indicate that customer mistreatment indirectly impacted frontline employees’ venting behaviors towards CAI, with emotional exhaustion functioning as the mediating mechanism. This indirect effect is particularly pronounced when employees exhibit high levels of trust in CAI. These findings offer practical insights for hospitality organizations aiming to leverage CAI as an accessible, low-risk tool for supporting employee emotional well-being and mitigating the negative consequences of customer mistreatment.

## 1. Introduction

Artificial intelligence (AI) is widely applied across the tourism and hospitality sectors. For instance, service robots assist frontline employees in performing various tasks, such as delivering meals to guests and supporting room cleaning (Bakir et al., 2025; Kumawat et al., 2025; Xu et al., 2023). Consequently, numerous studies in the hospitality field have examined collaboration between human frontline employees and service robots (Bai & Zhang, 2025; Qiu et al., 2022). Despite this burgeoning research on AI’s functional value in the hospitality industry, a critical research gap remains: existing studies have focused almost exclusively on AI’s role in task collaboration with employees or direct customer service, with no attention paid to conversational AI (CAI)’s potential as a tool for frontline employees themselves to regulate their negative emotional states triggered by workplace stressors. CAI, defined as digital agents capable of seamless interaction with users through natural language communication (P. Hu et al., 2021), has emerged as a promising system through which frontline employees may regulate negative emotional states. Venting to CAI represents an emotion-focused coping strategy whereby individuals discharge negative affective states by verbally or textually expressing their feelings to CAI systems (Erza et al., 2018; Gabriel et al., 2025; Rosen et al., 2021).

In the hospitality industry, frontline employees require an outlet to vent their negative emotions, primarily because they frequently face customer mistreatment (Guan et al., 2022; Huang & Kwok, 2021; Jiang et al., 2023; D. W. Liu et al., 2024). Customer mistreatment refers to disrespectful or abusive interpersonal treatment by customers during service interactions (Fan et al., 2022; M. Wang et al., 2011). Such behaviors may result in affective rumination (D. W. Liu et al., 2024), negative affect (Wong & Pan, 2023), and job burnout (H. B. Wang et al., 2022) among employees. Extant research on customer mistreatment has identified a range of employee coping strategies, yet these strategies are all centered on interpersonal interactions (e.g., seeking support from colleagues, suppressing emotions for customer relations) or behavioral adaptation toward customers, with no exploration of technology-mediated, non-interpersonal coping channels such as CAI. Considering the widespread prevalence of customer mistreatment in the hospitality industry and CAI’s capacity to withstand employee venting, this study focuses on hospitality employees with practical CAI experience and proposes the following research questions: After experiencing customer mistreatment, do employees vent about negative work events to CAI? If so, what are the underlying mediating mechanisms and boundary conditions?

To address these questions, we draw on conservation of resources (COR) theory, which posits that resource loss leads individuals to experience depletion and engage in behaviors aimed at retaining, acquiring, and safeguarding their core resources (Hobfoll, 1989, 2001). Frontline employees’ emotional and psychological resources suffer direct depletion owing to customer mistreatment, resulting in a state of emotional exhaustion (Han et al., 2016; H. S. Hu et al., 2017; H. B. Wang et al., 2022). To mitigate further resource loss and attempt replenishment, emotionally exhausted employees are motivated to take action (Dogantekin et al., 2022; S. M. Liu et al., 2022; Teng et al., 2024). Unlike venting to colleagues and family members, which entails interpersonal risks and demands additional resources (Gabriel et al., 2025; Rosen et al., 2021; X. S. Zhu et al., 2025), CAI provides a safe, anonymous, and low-risk outlet for emotional venting (P. Hu et al., 2021). Notably, CAI’s empathetic responses function as an effective form of psychological resource replenishment (Brandtzaeg et al., 2022; Skjuve et al., 2021; Song et al., 2022), enabling employees to acquire new resources necessary to cope with subsequent work tasks. Therefore, drawing on COR theory, this study conceptualizes venting to CAI as a resource-replenishing coping strategy triggered by customer mistreatment, while examining emotional exhaustion’s mediating role.

Furthermore, we consider trust in CAI as a key contingency factor, based on insights from COR theory, which proposes that individuals select channels for resource protection and acquisition when they perceive substantial resource gains and low potential risks associated with those channels (Hobfoll, 2001, 2011; Hobfoll et al., 2018). Trust in CAI refers to the extent to which frontline employees believe that CAI exhibits reliability, consistency, and confidentiality and can communicate smoothly with humans (Brunswicker et al., 2025; Chattaramana et al., 2019). Frontline employees’ trust in CAI reflects their cognitive evaluation of the benefits and risks inherent in this emotional venting channel. Higher levels of trust in CAI among frontline employees strengthen the intensity of their emotional venting to CAI when they experience emotional exhaustion.

This study makes several contributions to the literature. First, this study broadens the perspective on AI’s application to research on frontline employees in the hospitality industry. Overall, numerous studies have demonstrated the role of service robots in assisting frontline employees with customer service in the hospitality industry (Bakir et al., 2025; Gursoy et al., 2023; Kumawat et al., 2025; Xu et al., 2023), while others have elucidated CAI’s ability to directly communicate with customers and resolve their issues (Chandra et al., 2022; Corte et al., 2023; Pillai & Sivathanu, 2020; Y. Zhu et al., 2023); however, no study thus far has investigated how CAI, as a means for frontline employees to vent their emotions, can help these employees regulate their emotional states. Grounded in the context of frontline employees’ emotional venting, this study reveals the emotional value of venting to CAI, thus offering a novel perspective for future research.

Second, this study extends research on coping strategies for customer mistreatment. Existing studies have identified varied coping strategies, such as actively reinterpreting customer mistreatment behaviors (Niven et al., 2013), suppressing negative emotions (Wegge et al., 2010), engaging in surface acting (Nguyen & Stinglhamber, 2020), and adopting customer sabotage as a response (Ma et al., 2025; M. Wang et al., 2011). Nevertheless, the value of emerging technologies, such as CAI, in alleviating negative emotions caused by customer mistreatment remains unexplored. This study conceptualizes venting to CAI as a coping strategy for dealing with customer mistreatment, shifting the focus from interpersonal and customer-directed responses to a technology-mediated and inward-focused outlet. We posit that venting to CAI constitutes a distinct coping strategy that enables employees to manage the emotional toll associated with mistreatment through private, AI-facilitated disclosure rather than through behavioral adaptation towards customers or cognitive reappraisal of the event itself.

Third, this study advances the theoretical development of the literature on employee venting. Existing research has gradually evolved from the initial single venter-centric perspective to the venter–recipient interaction perspective, focusing on employee venting’s negative impacts on targets such as colleagues and leaders (Gabriel et al., 2025; Gong & Lee, 2026; D. W. Liu et al., 2024; Rosen et al., 2021). However, traditional venting research remains centered on interpersonal targets and has not yet recognized that CAI provides employees with a new venting channel that is low-cost, highly immediate, and free from social pressure. This study proposes and validates the behavioral pattern of employees venting to CAI, expanding venting channels from traditional offline interpersonal interaction to human–AI interaction, thereby filling the theoretical gap of AI-based venting channels in the literature on employee venting.

## 2. Theoretical Background and Hypothesis Development

### 2.1. Conceptual Distinction of Key Emotion-Related Constructs

Venting, coping, emotional regulation, and technology-assisted disclosure constitute distinct yet interrelated strategies for managing distress. Venting centers on the outward expression of internal negative emotions such as anger, frustration, and sorrow, and is generally characterized by its expressive and immediate nature (Gabriel et al., 2025; Gong & Lee, 2026; Rosen et al., 2021). Emotional regulation refers to the efforts individuals exert to influence which emotions they experience, when they experience them, as well as how these emotions are perceived and expressed, including cognitive reappraisal and acceptance (Chi & Liang, 2013; Gross, 1998; Gross et al., 2006; Mauss et al., 2007). Coping is the broader set of cognitive and behavioral efforts aimed at managing a specific stressor, encompassing both problem-focused strategies (e.g., active coping and planning) and emotion-focused strategies (e.g., positive reinterpretation, venting) (Lariviere et al., 2025; Lazarus, 1993; Liang et al., 2019). Technology-Assisted Disclosure refers to the use of digital tools (such as social media, diary apps, anonymous forums, and instant messaging) to disclose personal emotions and experiences to others or public or semi-public audiences (Clark-Gordon et al., 2019; Joinson, 2001; Lin et al., 2014).

### 2.2. Customer Mistreatment and Emotional Exhaustion

Drawing on COR theory, this study proposes that customer mistreatment results in emotional exhaustion among frontline employees, defined as a prolonged state of physical and emotional depletion arising from the cumulative effects of excessive job demands and ongoing work-related adversities (Maslach et al., 2001). Further, COR theory posits that individuals possess finite psychological resources, categorized as objects, personal characteristics, conditions, and energies valued by them, and an instinct to protect and safeguard these resources; consequently (Hobfoll, 1989, 2001), when these resources are continually depleted or lost, individuals experience psychological stress and emotional exhaustion (Chen & Eyoun, 2021; S. M. Liu et al., 2022; Pu et al., 2022).

In the hospitality industry, customer mistreatment is a highly prevalent work stressor and a typical work adversity for frontline employees, consuming a substantial portion of their emotional resources (Fan et al., 2022; Guan et al., 2022; Lan et al., 2022). When customers engage in verbal abuse, make unreasonable demands, or file complaints against frontline employees, the latter are required to adhere to organizational rules by suppressing negative emotions, such as anger or grievance, and feigning positive ones (Karatepe & Aleshinloye, 2009; H. B. Wang et al., 2022). This process of emotional regulation entails a continuous consumption of emotional resources, which precipitates emotional exhaustion (Karatepe & Aleshinloye, 2009; Li et al., 2017; H. B. Wang et al., 2022). Furthermore, when coping with unreasonable demands or conflicts from customers, frontline employees must mobilize cognitive resources to restrain their behaviors, for example, by remaining patient and maintaining a smiling demeanor while providing service (Pu et al., 2022; X. Wang & Wang, 2017). This process of self-control consumes substantial self-regulation resources, and the continual depletion of these resources tends to cause emotional exhaustion. Finally, customer mistreatment may directly undermine frontline employees’ sense of self-worth, adversely affecting their self-esteem (Huang & Kwok, 2021). As self-esteem is a critical psychological resource for coping with stress, its continuous impairment diminishes psychological resilience and further exacerbates emotional exhaustion among frontline employees (Huang & Kwok, 2021). Based on this evidence, we propose the following hypothesis:

**Hypothesis** **1 (H1).** *Customer mistreatment is positively associated with emotional exhaustion among frontline employees.*

### 2.3. Mediating Role of Emotional Exhaustion

Based on the above rationale, customer mistreatment arguably depletes a substantial portion of frontline employees’ psychological resources, potentially resulting in emotional exhaustion. According to COR theory, when confronted with resource depletion, individuals tend to proactively adopt resource protection or acquisition strategies to offset losses and restore resource balance (Halbesleben et al., 2014; Hobfoll, 1989, 2001). Thus, frontline employees experiencing emotional exhaustion may choose to vent to CAI as a resource compensation strategy.

Venting to CAI refers to the behaviors through which individuals express their emotions to CAI systems via verbal or written communication to discharge negative affective states (Erza et al., 2018; Gabriel et al., 2025; Rosen et al., 2021). Emotional exhaustion substantially depletes frontline employees’ emotional regulation resources, leaving them unable to manage negative emotions through internal self-regulation mechanisms (Hobfoll et al., 2018; Teng et al., 2024; H. Wang & Lee, 2026). Moreover, venting to colleagues and supervisors entails greater risks of resource consumption (Rosen et al., 2021; X. S. Zhu et al., 2025). Reportedly, venting to others typically yields only short-term mood-regulating effects and may even precipitate adverse consequences for venters, including intensified rumination and elevated stress levels (Brown et al., 2005; Nils & Rimé, 2012; X. S. Zhu et al., 2025). Furthermore, traditional channels may induce negative emotions among recipients, potentially triggering workplace conflicts and leader mistreatment (Rosen et al., 2021; X. S. Zhu et al., 2025). By contrast, CAI-based venting necessitates no additional investment of social resources, allowing frontline employees to freely express negative emotions, such as anger and dissatisfaction, through simple voice and text interactions (Leschanowsky et al., 2024; Mariani et al., 2023). Owing to its strong human-like characteristics, CAI can engage in emotional interaction with and comfort frontline employees, facilitating preliminary restoration of their emotional resources (Brunswicker et al., 2025; Corte et al., 2023; Mariani et al., 2023). Consequently, as frontline employees’ emotional exhaustion intensifies, their demand for low-cost, immediate resource compensation strategies grows, increasing their inclination to vent to CAI. In light of this argument, we propose the following hypotheses:

**Hypothesis** **2 (H2).** *Emotional exhaustion is positively associated with venting to CAI.*

Combining H1 and H2, we propose the following hypothesis:

**Hypothesis** **3 (H3).** *Emotional exhaustion mediates the relationship between customer mistreatment and venting to CAI.*

### 2.4. Moderating Role of Trust in CAI

Drawing on COR theory, this study proposes that the higher frontline employees’ trust in CAI, the more the pressure of emotional resource loss caused by emotional exhaustion motivates them to engage in emotional venting to CAI. Notably, COR theory holds that individuals generally weigh the potential resource gains and risks inherent in resource investment strategies when making their selection decisions (Hobfoll, 2001, 2011; Hobfoll et al., 2018). Trust in CAI reflects employees’ cognitive assessment of the benefits and risks associated with this emotional venting channel; these cognitive evaluations moderate the intensity of their emotional venting behaviors towards CAI when experiencing emotional exhaustion.

Trust in CAI refers to users’ positive beliefs formed during interactions with CAI systems regarding their reliability, consistency, confidentiality, and ability to communicate smoothly (Brunswicker et al., 2025; Chattaramana et al., 2019; Gefen et al., 2003; Pillai & Sivathanu, 2020). When frontline employees hold high levels of trust in CAI, they tend to perceive it as a reliable channel for regulating their emotional resources (Alimamy & Kuhail, 2023; Brunswicker et al., 2025; Pillai & Sivathanu, 2020). This cognitive perception elevates their expectations of resource gains from emotional venting to CAI, while simultaneously reducing their perceived potential risks of such behaviors, significantly strengthening emotional exhaustion’s driving effect on venting to CAI. Specifically, employees with high trust levels clearly perceive that emotional venting to CAI effectively helps them gain new emotional resources while carrying almost no risk of relational resource depletion owing to interpersonal venting (Brunswicker et al., 2025; Corte et al., 2023; Mariani et al., 2023; Rosen et al., 2021; X. S. Zhu et al., 2025). Consequently, trust in CAI further reinforces the positive relationship between employees’ level of emotional exhaustion and their engagement in venting to CAI.

When frontline employees hold low levels of trust in CAI, they tend to consider it an ‘unreliable venting channel’ (Alimamy & Kuhail, 2023; Chandra et al., 2022; Corte et al., 2023); hence, their expectations of resource gains from venting to CAI are relatively low, whereas their perceived potential risks are relatively high (Corte et al., 2023), weakening emotional exhaustion’s driving effect on venting to CAI. Even when employees experience high levels of emotional exhaustion, their motivation to engage in venting to CAI decreases because of their distrust of CAI (Alimamy & Kuhail, 2023; Chandra et al., 2022), significantly weakening emotional exhaustion’s positive effect on venting to CAI. Therefore, we propose the following hypothesis:

**Hypothesis** **4 (H4).** *The relationship between emotional exhaustion and venting to CAI is stronger when the level of trust in CAI is higher.*

**Figure 1:**
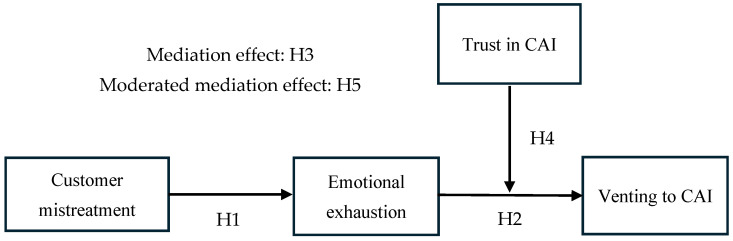
The conceptual model.

According to COR theory, individuals possess an inherent instinct to safeguard and preserve their psychological resources, and they experience emotional exhaustion when these resources are continually consumed or lost (Hobfoll, 1989, 2001). Meanwhile, individuals facing resource loss actively adopt resource acquisition strategies to restore resource balance (Halbesleben et al., 2014; Hobfoll, 1989, 2001). As mentioned earlier, emotional exhaustion mediates the relationship between customer mistreatment and venting to CAI. When frontline employees encounter verbal aggression, unreasonable demands, or complaints from customers, they are required to suppress negative emotions and feign positive emotions, which continuously consumes their emotional resources and precipitates emotional exhaustion. Frontline employees experiencing emotional exhaustion adopt resource compensation strategies and vent their emotions to CAI. If frontline employees perceive CAI as trustworthy, they are more likely to seek catharsis through CAI after experiencing emotional exhaustion caused by customer mistreatment. Therefore, we propose the final hypothesis, and Figure 1 displays our theoretical model.

**Hypothesis** **5 (H5).** *Trust in CAI moderates the indirect relationship between customer mistreatment and venting to CAI through emotional exhaustion, such that the relationship is stronger when the level of trust in CAI is higher.*

## 3. Methods

### 3.1. Sample and Procedures

We collected data using the Credamo platform, which has been employed in multiple studies to gather AI-related data from employees in the tourism and hospitality industry (Bakir et al., 2025). The study was conducted within the context of China’s hospitality industry, a sector characterized by high emotional labor and an increasingly digitized service environment (He et al., 2024; Yan et al., 2025). This context provides a theoretically significant setting for our research. In line with cultural norms that prioritize collective harmony over individual expression (Hofstede, 1980), Chinese service employees often suppress personal emotions during negative customer encounters to maintain organizational functioning, making China an ideal environment for examining how employees utilize CAI as a coping target for venting. We targeted frontline employees whose roles involve direct customer interaction to ensure that participants had sufficient exposure to potential customer mistreatment. Additionally, participants were required to have prior experience with CAI, ensuring that they were accustomed to venting work-related issues to such systems.

During the first wave, 686 questionnaires were administered; after excluding invalid responses, 550 valid responses were retained. Two weeks later, a second-wave questionnaire was administered among those who had completed the first wave, yielding 400 responses. After excluding responses that failed attention checks, 394 valid questionnaires remained. The final sample comprised 118 men (29.9%) and 276 women (70.1%). This gender distribution is consistent with prior research showing that women constitute a substantial proportion of the hospitality workforce in China, particularly in frontline service roles (e.g., Wu & Zhang, 2024; Yan et al., 2025). Participants’ average age was 32.8 years (SD = 7.27). Regarding educational background, 2.8%, 10.2%, 76.1%, and 10.9% of participants held a high school diploma or below, an associate degree, a bachelor’s degree, and a master’s degree or above, respectively. Participants’ average tenure at their current organization was 5.39 years (SD = 3.77).

### 3.2. Measures

We followed Brislin’s (1980) translation–back-translation procedure to translate all English scales into Mandarin Chinese.

Customer Mistreatment. Customer mistreatment was measured using an 8-item scale developed by Yue et al. (2017). At Time 1, participants were asked to indicate the extent to which they experienced mistreatment from customers using a 7-point scale (1 = very infrequently, 7 = very frequently); sample items include ‘My customer demanded special treatment’ and ‘My customer said inappropriate things’. The scale’s reliability was high, with Cronbach’s α = 0.92.

Emotional Exhaustion. Emotional exhaustion was assessed using a 9-item scale from Maslach and Jackson (1981), which measures feelings of being emotionally drained and overextended by one’s work. At Time 2, participants were asked to indicate the extent to which they experienced emotional exhaustion using a 7-point scale (1 = very infrequently, 7 = very frequently). Sample items include ‘I feel emotionally drained from my work’ and ‘I feel fatigued when I get up in the morning and have to face another day on the job’. The scale’s reliability was high, with Cronbach’s α = 0.94.

Trust in CAI. Trust in CAI was measured using a 4-item scale adapted from Pillai and Sivathanu (2020), which assesses the extent to which participants perceived chatbots as reliable and competent. At Time 2, participants were asked to report their levels of trust in CAI using a 7-point scale (1 = strongly disagree, 7 = strongly agree). Sample items include ‘I feel that information provided by chatbots is honest and authentic’ and ‘I feel chatbots are trustworthy’. The scale’s reliability was acceptable, with Cronbach’s α = 0.77.

Venting to CAI. Venting to CAI was measured using a 3-item scale adapted from Rosen et al. (2021), assessing the extent to which participants expressed negative work-related feelings to a CAI system. At Time 2, participants were asked to report their frequency of engaging in venting to CAI using a 7-point scale (1 = very infrequently, 7 = very frequently). Sample items include ‘I expressed anger about a work-related problem to a CAI’ and ‘I let out my negative feelings about work to a CAI’. The scale’s reliability was high, with Cronbach’s α = 0.93.

Control variables. We included control variables such as gender, age, company tenure, educational attainment, and AI use frequency.

## 4. Results

### 4.1. Confirmatory Factor Analysis

We conducted a series of confirmatory factor analyses (CFAs) to assess the study variables’ distinctiveness. Following Hair et al. (2019), we utilized CFA because our four primary constructs, customer mistreatment, emotional exhaustion, venting to conversational AI, and trust in conversational AI, are derived from established theories with clearly specified factor structures. Although we adapted the venting scale to reflect the technological context of this study, we maintained the conceptual core and functional purpose of the original construct. This adaptation simply specifies the target of the behavior (the conversational AI) without altering the underlying nature of the venting process. As presented in Table 1, the hypothesized four-factor model (customer mistreatment, emotional exhaustion, venting to CAI, and trust in CAI) exhibited satisfactory fit to the data ($\chi^{2}/df$ = 2.35, root mean square error of approximation = 0.06, comparative fit index = 0.95, Tucker–Lewis index = 0.94, standardized root mean square residual = 0.04). By contrast, several alternative three-factor models, wherein two constructs were combined, demonstrated significantly poorer fit. These results support the four focal constructs’ discriminant validity.

Furthermore, for convergent validity, the composite reliability (CR) values were 0.92 for customer mistreatment, 0.94 for emotional exhaustion, 0.93 for venting to conversational AI, and 0.77 for trust in CAI, all exceeding the recommended threshold of 0.70. The average variance extracted (AVE) values for customer mistreatment, emotional exhaustion, and venting to conversational AI were 0.60, 0.64, and 0.82, respectively, all above the 0.50 benchmark. Although the AVE for trust in CAI (0.46) fell slightly below 0.50, its CR (0.77) remained above the acceptable level, indicating adequate convergent validity. Taken together, these results suggest that the constructs demonstrate satisfactory reliability and unidimensionality. This approach is consistent with prior organizational research (e.g., Lopes et al., 2024; Verhoef et al., 2002). Finally, discriminant validity was supported using the Fornell-Larcker criterion: the square root of the AVE for customer mistreatment, emotional exhaustion, venting to conversational AI, and trust in CAI were 0.78, 0.80, 0.91, and 0.68, respectively, all of which exceeded its correlations with other constructs.

### 4.2. Descriptive Statistics

Table 2 presents the means, standard deviations, and correlations among the study variables. Customer mistreatment was positively correlated with emotional exhaustion (*r* = 0.37, *p* < 0.001) and venting to CAI (*r* = 0.42, *p* < 0.001), indicating that employees who experienced higher levels of mistreatment were more likely to feel emotionally drained and engage in venting behaviors. Additionally, emotional exhaustion was strongly associated with venting to CAI (r = 0.57, *p* < 0.001). Overall, the correlations aligned with theoretical expectations and provided preliminary support for the proposed relationships. To address potential common method bias, we conducted Harman’s single-factor test. An exploratory factor analysis of the 24 items yielded four factors with eigenvalues greater than 1.0. The first factor accounted for 38.03% of the total variance, which is well below the 50% threshold suggested by Podsakoff et al. (2003) and Malhotra et al. (2006). These results indicate that common method bias is not a significant concern in this study.

### 4.3. Hypothesis Testing

We tested our hypotheses using ordinary least squares regression analyses in R (Version 4.4.3). We mean-centered all predictor variables prior to analyses (Aiken et al., 1991). Results of the regression analyses further supported our hypotheses. H1 proposes that customer mistreatment is positively associated with emotional exhaustion. As presented in Model 2 of Table 3, customer mistreatment was positively associated with emotional exhaustion (*β* = 0.34, *p* < 0.001), thereby supporting H1.

H2 proposes that emotional exhaustion is positively associated with venting to CAI. In Model 4, emotional exhaustion was positively associated with venting to CAI (*β* = 0.64, *p* < 0.001), thereby supporting H2.

H3 proposes that emotional exhaustion mediates the relationship between customer mistreatment and venting to CAI. Customer mistreatment’s indirect effect on venting to CAI through emotional exhaustion was significant (indirect effect = 0.22, 95% confidence interval [CI] = [0.14, 0.30]), thereby supporting H3.

**Figure 2:**
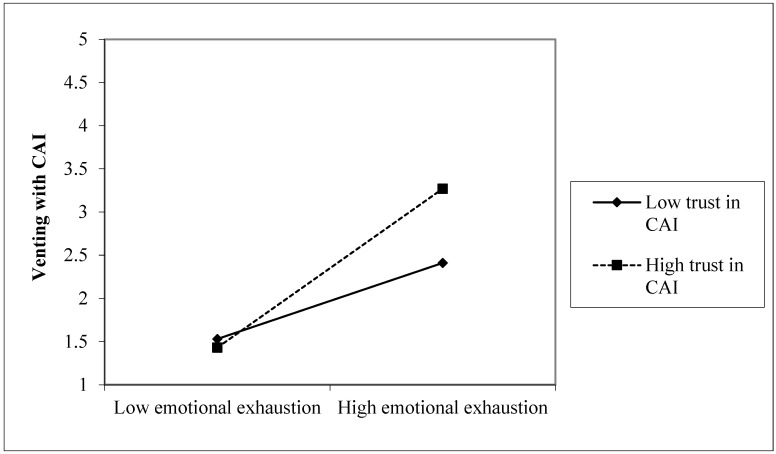
The moderating effect of trust in CAI.

H4 proposes that the relationship between emotional exhaustion and venting to CAI is stronger when the level of trust in CAI is higher. Consistent with H4, trust in CAI significantly moderated the relationship between emotional exhaustion and venting to CAI. In Model 5, the interaction term (Emotional exhaustion × Trust in CAI) was significant (*β* = 0.24, *p* < 0.001), indicating that emotionally exhausted employees were more likely to vent to CAI when their trust in CAI was higher. When trust in CAI was high (+1 SD), emotional exhaustion was strongly positively associated with venting to CAI (simple slope = 0.87, 95% CI = [0.71, 1.02]). When trust in CAI was low (−1 SD), emotional exhaustion remained positively associated with venting to CAI, though the effect was weaker (simple slope = 0.50, 95% CI = [0.36, 0.64]). The difference between the two slopes was significant (Δ = 0.37, 95% CI = [0.19, 0.55]). Figure 2 illustrates this moderating effect’s pattern.

H5 proposes that trust in CAI moderates the indirect relationship between customer mistreatment and venting to CAI through emotional exhaustion, such that the relationship is stronger when the level of trust in CAI is higher. As presented in Table 4, the moderated mediation analyses also revealed that customer mistreatment’s indirect effect on venting to CAI via through exhaustion was stronger at high levels of trust in CAI (effect_high = 0.29) rather than at low levels (effect_low = 0.17), with the difference being significant (difference = 0.12, 95% CI = [0.04, 0.21]), thereby supporting H5.

Overall, the results consistently support the hypothesized model, demonstrating that emotional exhaustion functions as a key mechanism whereby customer mistreatment leads employees to vent to CAI and that higher trust in CAI strengthens this mechanism.

### 4.4. Comparison of Results with Extant Literature

Prior studies have noted a consistent positive correlation between customer mistreatment and frontline employees’ emotional exhaustion (H. S. Hu et al., 2017; Pu et al., 2022; H. B. Wang et al., 2022), yet they have not explored the digital forms of resource-replenishing coping behaviors that employees may adopt to alleviate such exhaustion in the AI era. To explore this gap, our study empirically finds that venting to CAI acts as a common resource-replenishing coping behavior for frontline hospitality employees experiencing emotional exhaustion due to customer mistreatment, and further identifies the mediating role of emotional exhaustion in the link between customer mistreatment and employees’ venting to CAI through empirical analysis.

Furthermore, while previous research has primarily focused on interpersonal targets of employee venting, such as colleagues and supervisors (Gong & Lee, 2026; D. W. Liu et al., 2024; X. S. Zhu et al., 2025), our study introduces CAI as a novel and viable venting channel. The positive association between emotional exhaustion and venting to CAI supports the view that employees seek low-risk, immediate, and non-judgmental emotional outlets when facing resource depletion, yet such emotional release channels have been largely overlooked in existing literature on venting. This finding extends the research of Gabriel et al. (2025) and Rosen et al. (2021) by demonstrating that emotional venting need not be limited to human recipients, and that CAI can likewise serve as an effective channel for emotional release.

Finally, prior research on employee venting has identified a range of individual and contextual boundary conditions (e.g., leader-member exchange, organizational climate) (Gabriel et al., 2025; Rosen et al., 2021), but it has not examined the potential moderating role of trust in technology within the venting process. Our study reveals that trust in CAI significantly moderates the relationship between emotional exhaustion and venting to CAI, and a significant moderated mediation effect is identified. These findings contribute new empirical evidence to the limited literature on the boundary conditions of CAI-mediated venting behavior.

## 5. Discussion

This study revealed that emotional exhaustion mediated the relationship between customer mistreatment and venting to CAI among frontline employees. Further, trust in CAI was found to amplify customer mistreatment’s impact on venting to CAI through emotional exhaustion.

### 5.1. Theoretical Implications

This study makes several important theoretical contributions, as outlined below. First, it provides a novel perspective for research on CAI in the hospitality industry. Current interdisciplinary research at the intersection of hospitality management and human–AI interaction has predominantly focused on human–AI task collaboration, emphasizing AI’s functional value in assisting employees to optimize service processes (Bakir et al., 2025; Chandra et al., 2022; Corte et al., 2023; Kumawat et al., 2025; Pillai & Sivathanu, 2020; Y. Zhu et al., 2023); meanwhile, limited attention has been paid to CAI’s role in alleviating employees’ negative emotions. Grounded in the context of the hospitality industry, this study integrates customer mistreatment, emotional exhaustion, and trust in CAI to systematically clarify the triggering mechanism and reinforcing conditions of frontline employees’ venting to CAI. In doing so, it offers a new direction for future research and fosters theoretical integration and interdisciplinary innovation between hospitality management and AI.

Second, this study advances the development of research on coping strategies for customer mistreatment. Previous studies have found that employees’ strategies for coping with customer mistreatment have been largely limited to interpersonal-level coping and interventions, including actively reinterpreting customer mistreatment behaviors (Niven et al., 2013), suppressing negative emotions (Wegge et al., 2010), adopting surface acting (Nguyen & Stinglhamber, 2020), and responding with customer sabotage (Ma et al., 2025; M. Wang et al., 2011). This study extends the coping target to CAI, a non-interpersonal channel, thereby overcoming the ‘interpersonally oriented’ strategic limitations of this research field. Additionally, as a core theory explaining the transmission mechanism of workplace stress and negative behaviors, COR theory’s traditional application has predominantly focused on interpersonal interaction scenarios (Fan et al., 2022; Halbesleben et al., 2014; Halbesleben et al., 2014; Huang & Kwok, 2021). This study demonstrates that customer mistreatment triggers frontline employees’ venting to CAI through emotional exhaustion, suggesting that CAI tools may function as an emotional release outlet for employees following resource loss. This finding enhances COR theory’s applications across digital work scenarios and responds to the demand for expanding traditional theories with the development of digital technologies.

Third, this study contributes to the literature on venting by demonstrating that employees who experience customer mistreatment vent to CAI, which functions as a means for employees to regulate negative emotions (D. W. Liu et al., 2024); nevertheless, research on workplace venting has reported that listeners (i.e., individuals who receive venting) also suffer numerous negative outcomes, including negative emotions, psychological distress, and even uncivil behaviors towards colleagues and customers (Gabriel et al., 2025; Gong & Lee, 2026; Rosen et al., 2021; X. S. Zhu et al., 2025). Therefore, managers typically caution employees against venting at work; some organizations have even punished employees for venting (Robinson & Schabram, 2016; X. S. Zhu et al., 2025). However, negative events generate intense negative emotions among employees, and the failure to release these emotions negatively impacts their physical and mental well-being, as well as job performance (Park & Kim, 2020; Tao et al., 2019; H. B. Wang et al., 2022; Wong & Pan, 2023). Existing research has not recognized CAI as a novel channel for employees to engage in venting. This study proposes and validates the behavioral pattern in which employees vent to CAI, expanding venting channels from traditional ‘offline interpersonal interaction’ to ‘human–CAI interaction’ and filling the theoretical gap in the venting literature concerning CAI as an emerging venting channel.

Fourth, our theorizing regarding trust in CAI represents a distinct conceptual contribution to the literature on technology-mediated coping. We introduce trust in CAI as a critical boundary condition that clarifies when employees are likely to turn to a digital agent following real-world negative encounters (e.g., customer mistreatment), shifting the focus from whether venting to AI can occur to the conditions under which it becomes a probable and meaningful coping pathway. This shift deepens the current understanding of human–AI interaction in emotional contexts, suggesting that the mere availability of a CAI channel is insufficient. Rather, its perceived relational security, reflected in employees’ trust in CAI, determines whether it transforms from a passive tool into an active confidant within employees’ coping repertoires.

### 5.2. Practical Implications

First, this study’s findings enable frontline employees to recognize that they can vent to CAI to replenish their psychological energy when experiencing emotional exhaustion. For frontline employees facing customer mistreatment, venting to colleagues may incur additional personal resource costs (Brown et al., 2005; Nils & Rimé, 2012; X. S. Zhu et al., 2025), venting to relatives and friends may transmit negative emotions to others (Rosen et al., 2021; X. S. Zhu et al., 2025), and suppressing emotions internally may exacerbate emotional exhaustion (H. B. Wang et al., 2022). Against this background, CAI, owing to its capacity for real-time interaction, empathetic engagement, and robust privacy safeguards (Aw et al., 2022; B. Hu et al., 2023), is uniquely positioned to satisfy frontline employees’ needs for emotional release. Further, CAI offers a convenient, low-risk outlet for emotional expression, allowing them to vent negative feelings triggered by customer mistreatment at their convenience, whether during brief work breaks or after work hours, thereby discharging mental strain and alleviating emotional exhaustion. Additionally, this study demonstrates that employees’ trust in CAI functions as a critical moderating variable in the relationship between emotional exhaustion and venting to CAI. Specifically, a high level of trust increases employees’ willingness to release negative emotions through CAI, thereby alleviating emotional pressure. This finding provides valuable insights for enterprises seeking to optimise the application of CAI. Enterprises developing CAI tools should prioritise enhancing the system’s capabilities in safeguarding privacy and security and strengthening emotional recognition and response mechanisms (Alimamy & Kuhail, 2023; Corte et al., 2023), thereby enhancing both employees’ trust in CAI and CAI products’ market competitiveness (Alimamy & Kuhail, 2023; Corte et al., 2023; Pillai & Sivathanu, 2020). Furthermore, clarifying CAI’s positioning in supporting employee emotional management is essential. Finally, CAI should function as an auxiliary tool for emotional catharsis rather than as a primary substitute for human care, preventing employees from over-relying on CAI and disregarding genuine interpersonal communication.

### 5.3. Limitations and Future Research Directions

Despite this study’s contributions to the literature, it has several noteworthy limitations. First, the study data were self-reported by frontline employees, raising concerns regarding potential common method bias (Podsakoff et al., 2012). To mitigate the impact of this bias, this study adopted a longitudinal research design, with data collected at two time points separated by a two-week interval. Furthermore, this study sought to capture employees’ psychological states and subsequent venting to CAI, for which self-report measures are generally more accurate than other-report measures (Lan et al., 2022). Nevertheless, future studies should address this constraint by collating data from multiple sources, such as evaluations from supervisors and colleagues.

Second, this study’s sample exclusively comprised Chinese service employees, possibly limiting the findings’ generalizability. As cultures vary across countries, employees from diverse cultural backgrounds exhibit distinct psychological states and behavioral responses when facing customer mistreatment (Hofstede, 1980). Chinese culture emphasizes prioritizing collective interests over personal feelings (Hofstede, 1980); consequently, Chinese employees frequently suppress their individual emotions to safeguard collective benefits when experiencing customer mistreatment. Furthermore, CAI’s adoption has become widespread in other countries, including the United States (Drouin et al., 2022) and Thailand (Pal et al., 2023). Future studies should incorporate samples from diverse cultural contexts to expand on this study’s conclusions, thereby enhancing the findings’ generalizability. Furthermore, this study did not differentiate between employees’ hierarchical levels or functional departments. While our sample focused on customer-facing roles with direct CAI experience, the impact of customer mistreatment may vary across organizational strata. For instance, frontline staff often face more frequent interpersonal stressors than middle management. Similarly, employees in high-pressure areas (e.g., front office) may rely more heavily on CAI’s functional support than those in relatively shielded roles (e.g., housekeeping). Future research should adopt a more granular sampling approach to examine how job characteristics inherent to different positions and departments moderate the effectiveness of CAI.

Third, our use of a general measure for customer mistreatment may overlook the distinct effects of specific behaviors. Customer mistreatment is a multifaceted construct ranging from verbal abuse to unreasonable demands or service jaywalking. While an aggregate measure provides a holistic view of stressor intensity, different types of mistreatment may trigger unique psychological mechanisms, such as moral outrage versus resource depletion. We encourage future studies to utilize multidimensional scales or qualitative methods to explore whether CAI’s restorative role varies depending on the specific nature of the customer’s negative behavior.

Finally, beyond the moderator variable proposed in this study, namely, trust in CAI, future research should investigate CAI’s distinct characteristics, such as anthropomorphism level, emotional intelligence, perceived usefulness, and social influence (Corte et al., 2023; Pillai & Sivathanu, 2020; Y. Zhu et al., 2023). Future research should investigate in depth whether CAIs with varying levels of these attributes moderate the relationship between customer mistreatment and employees’ venting to CAI. Moreover, given the wide variety of CAI types available in the market, including intelligent assistants and chatbots (Chandra et al., 2022; Jiang et al., 2023; Song et al., 2022), future studies should systematically examine how different CAI types vary in their effectiveness in replenishing employees’ psychological resources.

## Figures and Tables

**Table 1:** Confirmatory factor analysis.

Models	χ^2^/df	RMSEA	CFI	TLI	SRMR
Four factor model: customer mistreatment, emotional exhaustion, venting to conversational AI, trust in conversational AI.	2.35	0.06	0.95	0.94	0.04
Three factor model: combination of customer mistreatment and venting to conversational AI.	5.97	0.11	0.81	0.79	0.11
Three factor model: combination of venting to conversational AI and trust in conversational AI.	6.90	0.12	0.78	0.75	0.16
Three factor model: the combination of customer mistreatment and emotional exhaustion.	8.89	0.14	0.70	0.67	0.14

**Table 2:** Means, standard deviations and correlations between variables.

	Mean	SD	1	2	3	4	5	6	7	8	9
1. Customer mistreatment	3.10	1.16									
2. Emotional exhaustion	2.68	1.16	0.37 ***								
3. Venting to conversational AI	3.14	1.49	0.42 ***	0.57 ***							
4. Trust in conversational AI	5.66	0.76	−0.11 *	−0.23 ***	−0.05						
5. Gender ^a^	0.70	0.46	0.06	0.13 **	0.03	−0.13 *					
6. Age	32.80	7.27	−0.13 *	−0.26 ***	−0.17 ***	0.04	0.08				
7. Education	3.96	0.62	0.13 *	−0.02	0.08	0.06	−0.05	0.05			
8. Company tenure	5.39	3.77	−0.07	−0.26 ***	−0.13 **	0.09	0.04	0.73 ***	0.11 *		
9. AI use frequency ^b^	4.52	0.56	0.00	−0.16 **	−0.05	0.19 ***	−0.05	0.01	0.05	0.04	

Note: N = 394; * *p* < 0.05, ** *p* < 0.01 *** *p* < 0.001; ^a^ Employee gender: 0 = male, 1 = female. ^b^ AF: 1 = almost never, 2 = seldom use, 3 = several times a month, 4 = several times a week, 5 = almost every day.

**Table 3:** The results of the regression analysis.

	Emotional Exhaustion		Venting to Conversational AI		
	M1	M2	M3	M4	M5
Constant	4.77 *** (0.65)	4.88 *** (0.61)	3.90 *** (0.87)	2.66 *** (0.71)	2.91 *** (0.69)
*Control variable*					
Gender	0.37 ** (0.12)	0.30 ** (0.11)	0.14 (0.16)	−0.15 (0.13)	−0.15 (0.13)
Age	−0.03 * (0.01)	−0.02 (0.01)	−0.03 * (0.01)	−0.01 (0.01)	−0.01 (0.01)
Education	0.03(0.09)	−0.05 (0.09)	0.23 (0.12)	0.13 (0.10)	0.17 (0.10)
Company tenure	−0.04 (0.02)	−0.04 * (0.02)	−0.01 (0.03)	0.01 (0.02)	0.01 (0.02)
AI use frequency	−0.30 ** (0.10)	−0.30 ** (0.09)	−0.14 (0.13)	0.05 (0.11)	−0.02 (0.11)
*Independent variable*					
Customer mistreatment		0.34 *** (0.05)		0.29 *** (0.06)	0.29 *** (0.05)
*Mediator*					
Emotional exhaustion				0.64 *** (0.06)	0.68 *** (0.06)
*Moderator*					
Trust in conversational AI					0.19 * (0.08)
Emotional exhaustion * Trust in conversational AI					0.24 *** (0.06)
*R* ^2^	0.12 ***	0.23 ***	0.04 **	0.39 ***	0.42 ***

Note: *N* = 394; * *p* < 0.05, ** *p* < 0.01, *** *p* < 0.001. Unstandardized regression coefficients are displayed, with standard errors in parentheses.

**Table 4:** Moderated mediation analyses.

Moderator: Trust in CAI	Effect	SE	LLCI	ULCI
Path: customer mistreatment → emotional exhaustion → venting with conversational AI				
Low (−1 SD)	0.17	0.04	0.10	0.25
High (+1 SD)	0.29	0.05	0.20	0.40
Difference	0.12	0.04	0.04	0.21

Note: *N* = 394.

## Data Availability

Dataset available on request from the authors.
